# E2F1-driven EXOSC10 transcription promotes hepatocellular carcinoma growth and stemness: a potential therapeutic target

**DOI:** 10.1186/s41065-025-00430-7

**Published:** 2025-04-12

**Authors:** Haoyue Deng, Dingyong Wu, Yongpeng He, Xiaolei Yu, Jifei Liu, Yanrui Zhang, Bing Leng, Xiaofeng Yuan, Liguo Xiao

**Affiliations:** 1 Department of Pathology, Suining Central Hospital, Suining, 629000 Sichuan China; 2 Department of Oncology, Songshan General Hospital, Chongqing, 401120 China; 3 Chongqing Key Laboratory of Translational Research for Cancer Metastasis and Inaffiliationidualized Treatment, Chongqing University Cancer Hospital & Chongqing Cancer Institute & Chongqing Cancer Hospital, Chongqing, 400030 China; 4 Department of Oncology, Fengning Manchu Autonomous County Hospital, No.737 Binhe Road, Chengde, 067000 Hebei China

**Keywords:** E2F1, EXOSC10, HCC, Stemness, Angiogenesis, Proliferation

## Abstract

**Background:**

E2F Transcription Factor 1 (E2F1) is a transcription factor that plays a crucial role in the growth of many cancers, including hepatocellular carcinoma (HCC). Herein, this study probed the functions and underlying mechanisms of E2F1 in HCC tumorigenesis.

**Methods:**

The expression profiles of E2F1 and Exosome Component 10 (EXOSC10) were detected using qRT-PCR and western blotting. Functional experiments were carried out using 5-ethynyl-2’-deoxyuridine (EdU), flow cytometry, tube formation, and sphere formation assays in vitro, as well as xenograft experiments in vivo, respectively. Stemness-related proteins were assayed using western blotting. The interaction between E2F1 and EXOSC10 was verified using bioinformatics analysis and dual-luciferase reporter assay.

**Results:**

E2F1 was highly expressed in HCC tissues and cells, and was associated with advanced TNM stage, distant metastasis, and short survival rate. Functionally, knockdown of E2F1 suppressed HCC cell proliferation, angiogenesis, and stemness, and induced cell apoptosis. Mechanistically, E2F1 directly bound to the promoter region of EXOSC10 to up-regulate its expression. EXOSC10 silencing impaired HCC cell proliferation, angiogenesis, and stemness. Moreover, the anticancer effects of E2F1 knockdown were reversed by EXOSC10 elevation. In vivo assay, E2F1 deficiency suppressed HCC tumor growth and eliminated cancer stemness, while these effects were abolished by EXOSC10 up-regulation.

**Conclusion:**

E2F1 promotes EXOSC10 transcription and then facilitates HCC growth and cancer stemness, revealing a potential target for HCC therapy.

**Supplementary Information:**

The online version contains supplementary material available at 10.1186/s41065-025-00430-7.

## Introduction

Hepatocellular carcinoma (HCC) is the most common type of liver cancer, and there are 865,269 new cases and 757,948 deaths worldwide in 2022 [1, 2]. Chronic hepatitis caused by the infection of hepatitis B and hepatitis C viruses is an important risk factor for HCC, besides that, alcohol and fat consumption in the liver also increases the risk of HCC [3]. Although a broad range of treatment options have been developed, including surgical resection, liver transplantation, percutaneous ablation and radiation, as well as transarterial and systemic therapies, the 5-year overall survival rate of HCC patients is still unsatisfactory owing to the high frequency of recurrent distant metastases and drug resistance [4–8]. Therefore, further investigation on the pathogenesis of HCC is necessary for developing effective therapeutic strategy for HCC patients.

Currently, epigenetic alterations have been revealed to contribute to the development of HCC by affecting the gene expression [5, 9]. E2F Transcription Factor 1 (E2F1) is a transcription factor that plays a crucial role in controlling cell cycle, apoptosis, DNA-damage response, and senescence in physiological and pathological conditions [10–12]. Interestingly, the dysregulation of E2F1 is known to be involved in the tumorigenesis. For example, Jing et al. showed that Non-SMC Condensin II Complex Subunit D3 (NCAPD3) bound to E2F1 to increase E2F1-mediated transcription of Pyruvate dehydrogenase kinase (PDK) 1 and PDK3 genes, then suppressed the pyruvate dehydrogenase activity and tricarboxylic acid (TCA) cycle, thereby promoting colorectal cancer progression by enhancing Warburg effect [13]. E2F1 was a target of chondroitin polymerizing factor (CHPF), and silencing of E2F1 abolished CHPF-induced growth and migration in gastric cancer [14]. Phosphatase and tensin homolog (PTEN) could bind to E2F1 to suppress E2F1-mediated transcription, and then impaired cell cycle in lung cancer [15]. Importantly, highly expressed E2F1 is tightly linked with the genesis and progression of HCC. E2F1 interacted with and regulated the transcription of miR-224-5p, which contributed to the metastasis of HCC cells [16]. Qiao’s team showed that Ubiquitin specific peptidase 11 (USP11) bound to E2F1 and promoted its stability by deubiquitination, and E2F1/USP11 formed a feedback loop to impede HCC tumor growth by inhibiting autophagy via activating ERK/mTOR pathway [17]. Centromere protein U (CENPU) promoted the growth and metastasis by maintaining E2F1 stability through removing E2F6 ubiquitin [18]. Thus, E2F1 inhibition may be involved in the prevention of HCC. In addition, the JASPAR database showed that E2F1 has two binding sites on the promoter region of Exosome Component 10 (EXOSC10), and the Starbase database showed that the expression of EXOSC10 is positively correlated with E2F1 in HCC tissues. EXOSC10, a 3’-5’exoribonuclease, is required for gametogenesis, erythropoiesis, brain development, and blood cell enhancer function [19]. Besides that, deregulated EXOSC10 has been confirmed to be involved in HCC progression [20]. However, the relationship between E2F1 and EXOSC10 in HCC remain unclear.

Here, this study focused on probing the functions of E2F1 and EXOSC10 on HCC growth and cancer stemness, moreover, their interaction in HCC progression were further investigated, which may provide a novel insight into the therapy of HCC.

## Materials and methods

### Samples collection

HCC tissues and adjacent normal tissues were collected during surgery from 66 HCC patients admitted at Suining Central Hospital. HCC patients were pathologically diagnosed and had not received any preoperative treatment. This study was authorized by the Ethics Committee of Suining Central Hospital.

### Cell culture

Huh-7, Hep3B, and normal THLE-2 cells were obtained from Procell (Wuhan, China). HUVECs were provided by ATCC (Manassas, VA, USA). Huh-7 and Hep3B, as well as HUVECs were cultivated in RPMI1640 medium (#12-167-F, Lonza, Basel, Switzerland) plus 1% penicillin/streptomycin (# PB180120, Procell, Wuhan, China) and 10% FBS (#A5670701, Gibco, Grand Island, NY, USA ). THLE-2 cells were cultured in BEGM Bullet Kit (#CC-3170, Lonza). All the medium was maintained in an incubator with 5% CO_2_ at 37℃.

### Cell transfection

Small interfering RNAs (siRNAs) targeting E2F1 (si-E2F1#1 or si-E2F1#2) or Exosome Component 10 (EXOSC10) (si-EXOSC10), pcDNA3.1-EXOSC10 overexpression plasmids and the controls (nontargeted siRNAs and empty pcDNA3.1 plasmids) (si-NC and pcDNA) were designed by GenePharma (Shanghai, China). Lentiviral plasmids carrying E2F1-specific short hairpin RNA (shRNA) (sh-E2F1) and a scrambled shRNA (sh-NC) using pLentiLox 3.7 plasmids were provided by Hanbio (Shanghai, China) for in vivo assay. Lipofectamine 3000 (#L3000150, Invitrogen, Carlsbad, CA, USA) was used for transfection following the protocols.

### Quantitative Real-Time PCR (qRT-PCR)

Total RNAs were extracted from tissues and cells using Trizol (#R401-01, Vazyme, Nanjing, China). Then the PrimeScript™ RT kit (#RR037Q, TaKaRa, Dalian, China) and TB Green^®^ Premix Ex Taq™ (#RR420Q, Takara) were used to generate cDNAs and conduct qRT-PCR analysis. Table 1 shows the primers.

**Table 1:** Primers sequences used for qRT-PCR

Name		Primers for qRT-PCR (5’-3’)
E2F1	Forward	AAACAAGGCCCGATCGATGT
	Reverse	TGGGATCTGTGGTGAGGGAT
EXOSC10	Forward	GTCTCTCAGGCAGCGAAGTT
	Reverse	CTGTTTGCTCAGCTGCCTTC
GAPDH	Forward	ATCACTGCCACCCAGAAGAC
	Reverse	CCGTTCAGCTCAGGGATGAC

### Western blotting

Tissues and cells were lysed using RIPA lysis buffer to isolate total proteins, and the concentration of proteins was quantified by an Enhanced BCA Protein Assay Kit (#P0010S, Beyotime, Beijing, China). 30 µg of protein were separated using an SDS-PAGE and then shifted onto nitrocellulose membranes as described before [21, 22]. After being sealed by 10% non-fat milk at 37℃for 2 h, primary antibodies against E2F1 (ab55325, 1:1000), EXOSC10 (ab94981, 1:1000), Cleaved Caspase-3 (ab2302, 1:500), Bcl-2 (ab182858, 1:2000), VEGFA (ab46154, 1:1000), FGF2 (ab208687, 1:1000), PDGF-A (ab51868, 1:1000), OCT4 (ab181557, 1:1000) and CD44 (ab157107, 1:2000) (Abcam, Cambridge, MA, USA) were applied to incubate with membranes overnight at 4℃. After washing, membranes were incubated with Goat Anti-Rabbit IgG H&L (ab205718) or Goat Anti-Mouse IgG H&L (ab205719) secondary antibody for 2 h at 37℃, followed by detecting protein bands using the BeyoECL Star (#P0018AS, Beyotime).

### EdU assay

Huh-7 and Hep3B cells were incubated for 2 h with 50 µM EdU solution (#C10310, RiboBio, Guangzhou, China) in a 96-well plate, followed by dyeing with 100 µL 1× Apollo^®^ staining reaction (#C10310, RiboBio) avoiding light for half anhour. Next, DAPI (#62248, Invitrogen) staining was used for cell nuclei. EdU-positive cells were captured and counted using an EVOS M5000 microscope (Thermo Fisher Scientific, Shanghai, China).

### Flow cytometry

Huh-7 and Hep3B cells were adjusted to single cell suspension of 1 × 10^6^ cells/mL with binding buffer. Then 100 µL cell suspension was used to incubate with staining buffer consisting of 5 µL Annexin V-FITC and 5 µL propidium iodide (#BB-4101, BestBio, Shanghai, China) for 15 min avoiding light. Lastly, cell apoptosis was determined using flow cytometry.

### Tube formation assay

A 96-well plate was added with 50 µL chilled Matrigel (#356234, BD Biosciences, Franklin Lakes, NJ, USA) in each well and then was incubated at 37℃ for 30 min to allow the basement membrane to gel. The conditioned medium (CM) of assigned Huh-7 and Hep3B cells was collected. HUVECs were serum-starved in RPMI1640 medium for 2 h. Then HUVECs (1 × 104/well) and 100 µL CM was added into a 96-well plate that was pre-coated with 50 µL chilled Matrigel. After 6 h incubation, tubular structures were observed and manually counted.

### Sphere formation assay

Huh-7 and Hep3B cells were incubated with serum-free medium in a 96 well plate. The medium was changed every 2 days. After 14 days incubation, sphere formation efficiency was calculated.

### Dual-luciferase reporter assay

The target fragments containing wild-type (wt) binding sites on EXOSC10 sequences and the mutated (mut) sequences were amplified and inserted into the *NotI* and *XhoI* of psiCHECK-2 vectors (#C8021, Promega, Beijing, China) to establish the EXOSC10-wt/mut vectors. Two hours before transfection, Huh-7 and Hep3B cells were harvested and cultured in fresh serum-free RPMI1640 medium. The 5 ug recombinant plasmids was diluted with 250 µL serum-free Opti-MEM, named A solution. 250 µL Opti-MEM culture solution and 6.0 µL Lipofectamine 2000 reagent were mixed, named B solution. Then the A and B solution was mixed and incubated at room temperature for 15 min. 100 µL mixture was added into a 6-well plate and incubated for 12 h. Then the culture medium was discarded and RPMI1640 medium containing 10% FBS was added. After further 24 h incubation, the luciferase activity was assayed.

### Xenograft mouse model

BALB/c nude mice (female, 4–5 weeks old, *n* = 18) were obtained from Slaike Jingda Laboratory (Hunan, China). Huh-7 cells transfected with sh-E2F1 or sh-NC were injected into each mice and then divided into three group (*n* = 6/group): sh-NC, sh-E2F1, or sh-E2F1 + EXOSC10 group. When tumors were about 100 mm^3^ in volume, mice in sh-E2F1 + EXOSC10 group were intratumorly injected with EXOSC10 vectors in Lipofectamine at 3-5 sites of the xenografts every 3 days. The subcutaneous tumor volume was measured at 7-day interval. At day 28, mice were anaesthetized and tumors were isolated and weighed. The animal experiment was approved by the Animal Research Committee of Suining Central Hospital.

### Immunohistochemistry (IHC) assay

Paraffin xenograft sections were dewaxed and hydrated, then treated with 3% H_2_O_2_ to quench endogenous peroxidase. Sections were blocked in 1% BSA for 30 min, and then hatched with Ki67 (ab15580, 1:1000, Abcam) at 4℃ all night. Then sections were hatched with goat anti-rabbit IgG H&L (HRP) (ab6721, 1:1000, Abcam) for 1 h at 37℃ after being washed with PBS. Subsequently, sections were stained with 3,3’-Diaminobenzidine (DAB) (#ST3205-5 g, Beyotime) and counterstained in hematoxylin solution (#C0107, Beyotime).

### Statistical analysis

The data were manifested as mean ± standard deviation (SD). Student’s *t*-test and one-way analysis of variance (ANOVA) were respectively employed to compare the difference of variables. Pearson’s correlation analysis was conducted for correlation analysis. Survival curves were generated using the Kaplan-Meier method with log-rank test. *P* < 0.05 denotes asignificant difference.

## Results

### E2F1 is highly expressed in HCC tissues and cells

**Fig. 1:**
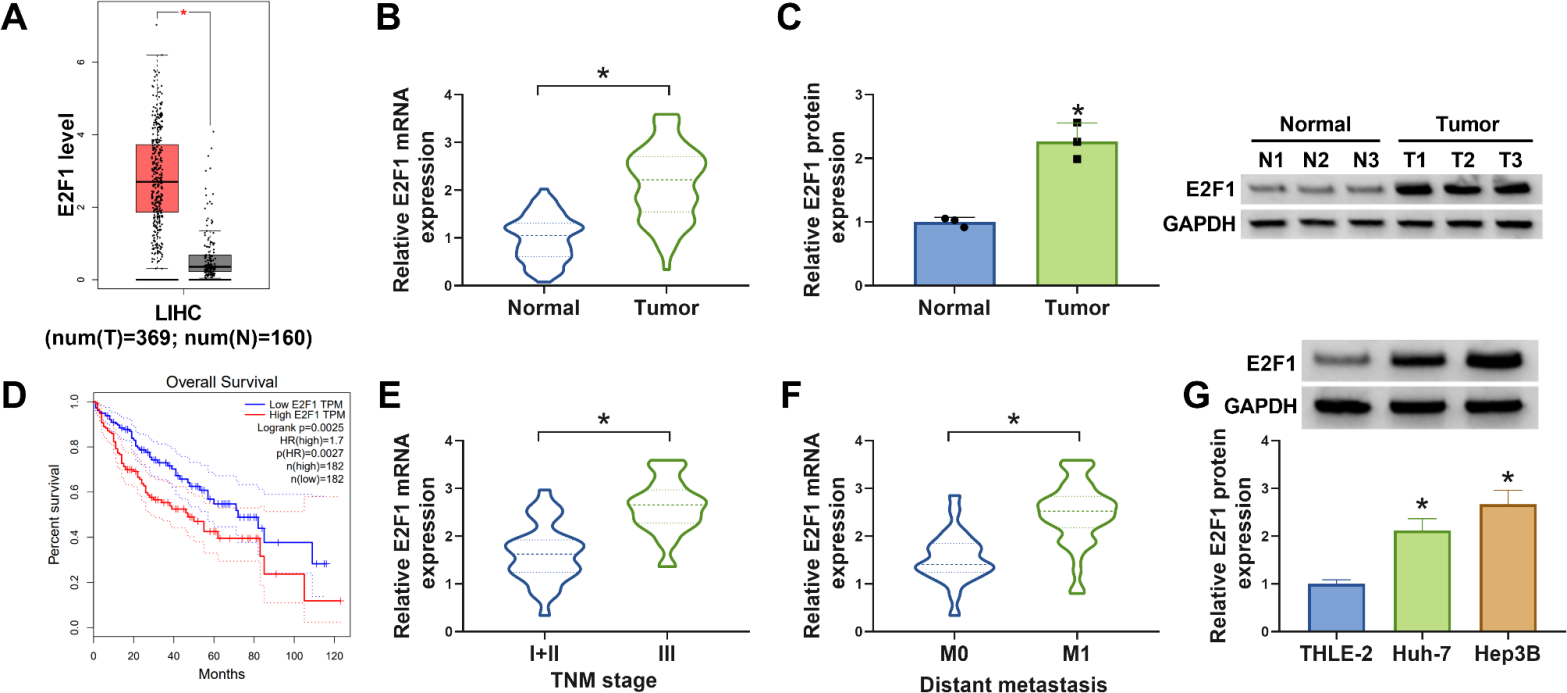
E2F1 is highly expressed in HCC tissues and cells. (**A**) GEPIA database shows the expression profiles of E2F1 in HCC. (**B, C**) qRT-PCR and western blotting analysis for E2F1 expression in HCC and normal tissues. (**D**) Kaplan-Meier survival curves of overall survival for HCC patients with high or low E2F1 expression. (**E, F**) qRT-PCR analysis for E2F1 expression in HCC tissues at different TNM stage or with/without distant metastasis. (**G**) Western blotting analysis for E2F1 expression in HCC cell lines or normal THLE-2 cells. *P < 0.05

According to the GEPIA database, the expression levels of E2F1 were higher in HCC tissues than those in normal tissues (Fig. 1A). Then the 66 clinical samples of HCC were collected. The clinicopathological characteristics of HCC patients have been provided in Table S1. As expected, E2F1 expression was elevated in HCC tissues at both mRNA (about 2.1 folds) and protein (about 2.3 folds) levels compared with the normal tissues (Fig. 1B, C). Moreover, patients with high E2F1 expression showed shorter survival rate compared with patients with low E2F1 level (Fig. 1D). In addition, E2F1 expression was higher in HCC patients at advanced stage (TNM III) relative to TNM I + II stage (Fig. 1E). And HCC patients with distant metastasis (M1) showed higher E2F1 level compared those with no distant metastasis (Fig. 1F). Also, we found an increased expression of E2F1 in HCC cell lines compared with normal THLE-2 cells (Fig. 1G).

### E2F1 Silencing suppresses HCC cell proliferation, angiogenesis, and stemness

**Fig. 2:**
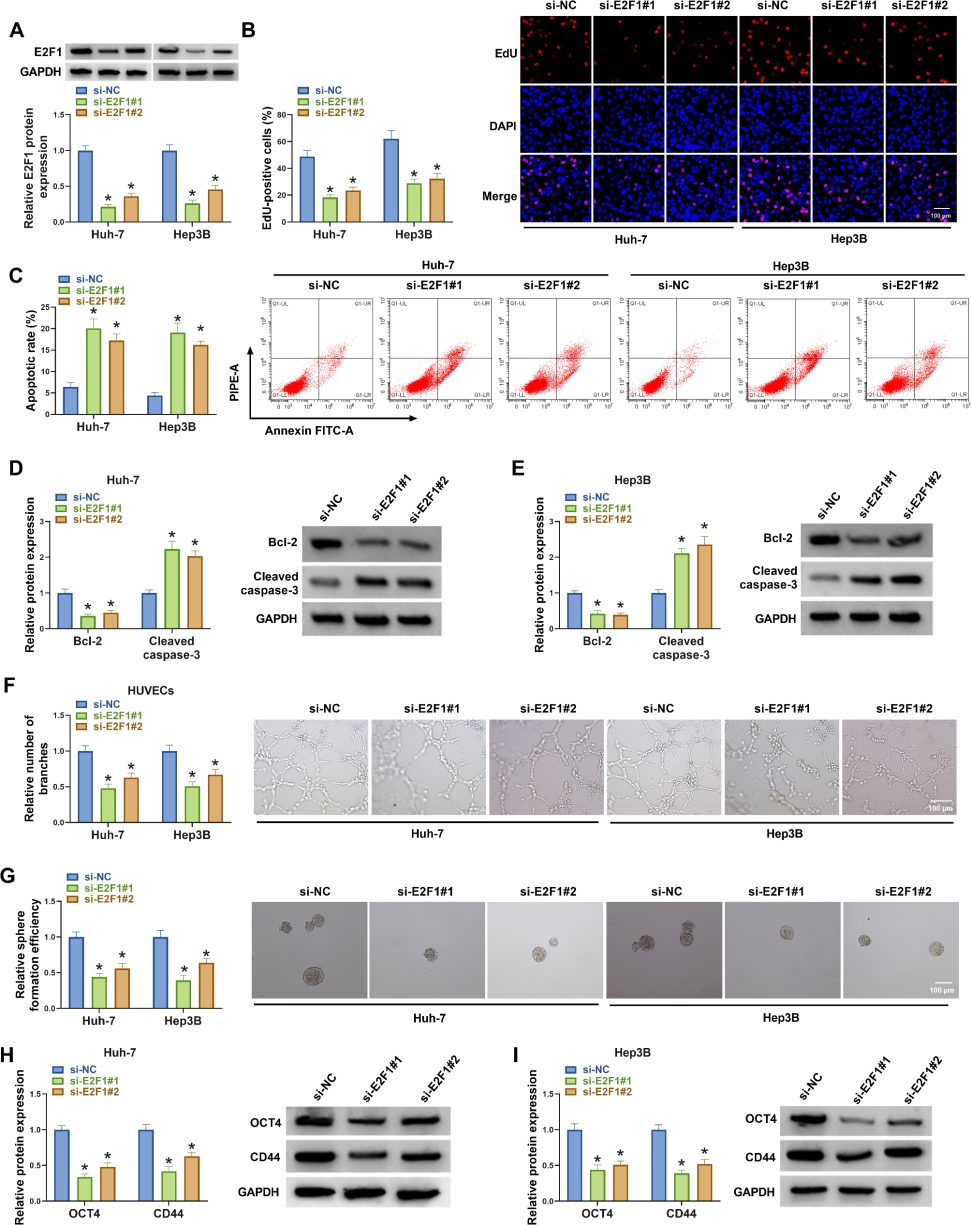
E2F1 silencing suppresses HCC cell proliferation, angiogenesis, and stemness. (**A-G**) Huh-7 and Hep3B cells were transfected with si-NC, si-E2F1#1, or si-E2F1#2. (A) Western blotting analysis for E2F1 expression in cells. (**B**) EdU assay for cell proliferation. (**C**) Flow cytometry for cell apoptosis. (**D, E**) Detection of Bcl-2 and Cleaved-caspase-3 protein levels by western blotting. (**F**) Analysis of capillary-like structures formed by HUVECs by tube formation assay. (**G**) Tumor sphere formation assay for sphere formation rate. (**H, I**) Detection of OCT4 and CD44 protein levels by western blotting. *P < 0.05

Thereafter, the action of E2F1 on HCC cell oncogenic phenotypes was investigated. The E2F1 siRNAs were designed, western blotting analysis showed that si-E2F1#1 led to about 5 folds reduction in Huh-7 cells and about 3 folds reduction in Hep3B cells, and si-E2F1#2 induced about 4 folds reduction in Huh-7 cells and about 2.5 folds reduction in Hep3B cells (Fig. 2A), indicating the successful interference of E2F1 in HCC cells. Functionally, E2F1 silencing reduced the number of EdU-positive Huh-7 and Hep3B cells (Fig. 2B), suggesting the inhibition of cell proliferation. But E2F1 silencing induced apoptosis in Huh-7 and Hep3B cells (Fig. 2C). Moreover, we observed that E2F1 silencing reduced the expression of anti-apoptotic Bcl-2 protein and increased the levels of Cleaved-caspase-3 protein in Huh-7 and Hep3B cells (Fig. 2D, E), further suggesting the promotion of cell apoptosis. In addition, E2F1 silencing suppressed the formation of capillary-like structures of HUVECs (Fig. 2F), suggesting the inhibition of tumor angiogenesis. Besides, angiogenesis-associated proteins were detected, and western blotting showed that E2F1 silencing reduced the expression of growth factors VEGFA, bFGF and PDGF-A, further suggesting the antiangiogenic role of decreased E2F1 (Fig. S1). The tumor sphere formation assay suggested that E2F1 deficiency decreased the sphere formation rate in Huh-7 and Hep3B cells (Fig. 2G). Further western blotting analysis manifested that E2F1 deficiency reduced the expression of OCT4 and CD44, which are key regulators of cancer stemness, in Huh-7 and Hep3B cells (Fig. 2H, I).

### E2F1 elevates EXOSC10 expression by promoting the transcription of EXOSC10

**Fig. 3:**
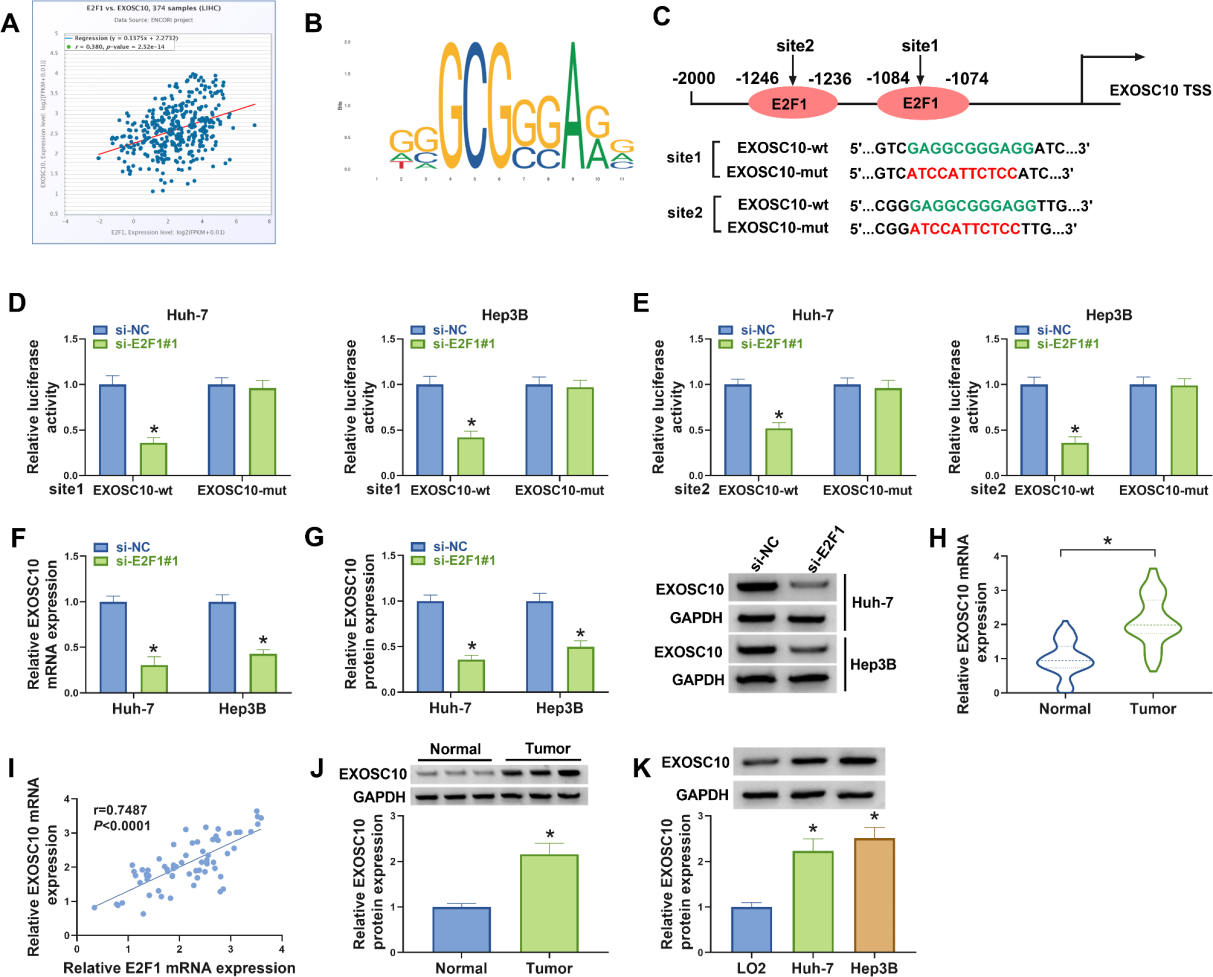
E2F1 elevates EXOSC10 expression by promoting the transcription of EXOSC10. (**A**) The Starbase database predicted that EXOSC10 expression was positively correlated with E2F1 in HCC tissues. (**B, C**) JASPAR database (https://jaspar.genereg.net/) showed that E2F1 has two binding sites on the promoter region of EXOSC10. (**D, E**) Dual-luciferase report assay was performed to probe the binding between E2F1 and EXOSC10. (**F, G**) qRT-PCR and western blotting analysis for EXOSC10 expression in Huh-7 and Hep3B cells after E2F1 knockdown. (**H**) qRT-PCR analysis for EXOSC10 mRNA expression in HCC tissues and normal tissues. (**I**) Correlation analysis between E2F1 and EXOSC10 in HCC tissues. (**J, K**) Western blotting analysis for EXOSC10 protein expression in HCC tissues and normal tissues, as well as in HCC cell lines or normal THLE-2 cells. *P < 0.05

The Starbase database predicted that the expression of EXOSC10 was positively correlated with E2F1 in HCC tissues (Fig. 3A). Further JASPAR database (https://jaspar.genereg.net/) showed that E2F1 has two binding sites on the promoter region of EXOSC10 (Fig. 3B, C). Then the relationship between EXOSC10 and E2F1 was explored. The two binding sites of E2F1 on EXOSC10 were mutated using mutagenesis kits, named as EXOSC10-mut. Next, dual-luciferase report assay showed that E2F1 knockdown notably reduced the luciferase activities of the wild-type EXOSC10 vectors at the both sites, but not the mutated one in Huh-7 and Hep3B cells (Fig. 3D, E), indicating the binding between E2F1 and EXOSC10. In addition, E2F1 silencing led to a decrease of EXOSC10 level in HCC cells (Fig. 3F, G). Thereafter, the expression profiles of EXOSC10 were investigated. We found EXOSC10 mRNA expression was increased in HCC tissues (Fig. 3H) and also positively correlated with E2F1 expression (Fig. 3I). Also, EXOSC10 protein levels were higher in HCC tissues (Fig. 3J) and cell lines (Fig. 3K) compared with the controls.

### EXOSC10 silencing suppresses HCC cell proliferation, angiogenesis, and stemness

**Fig. 4:**
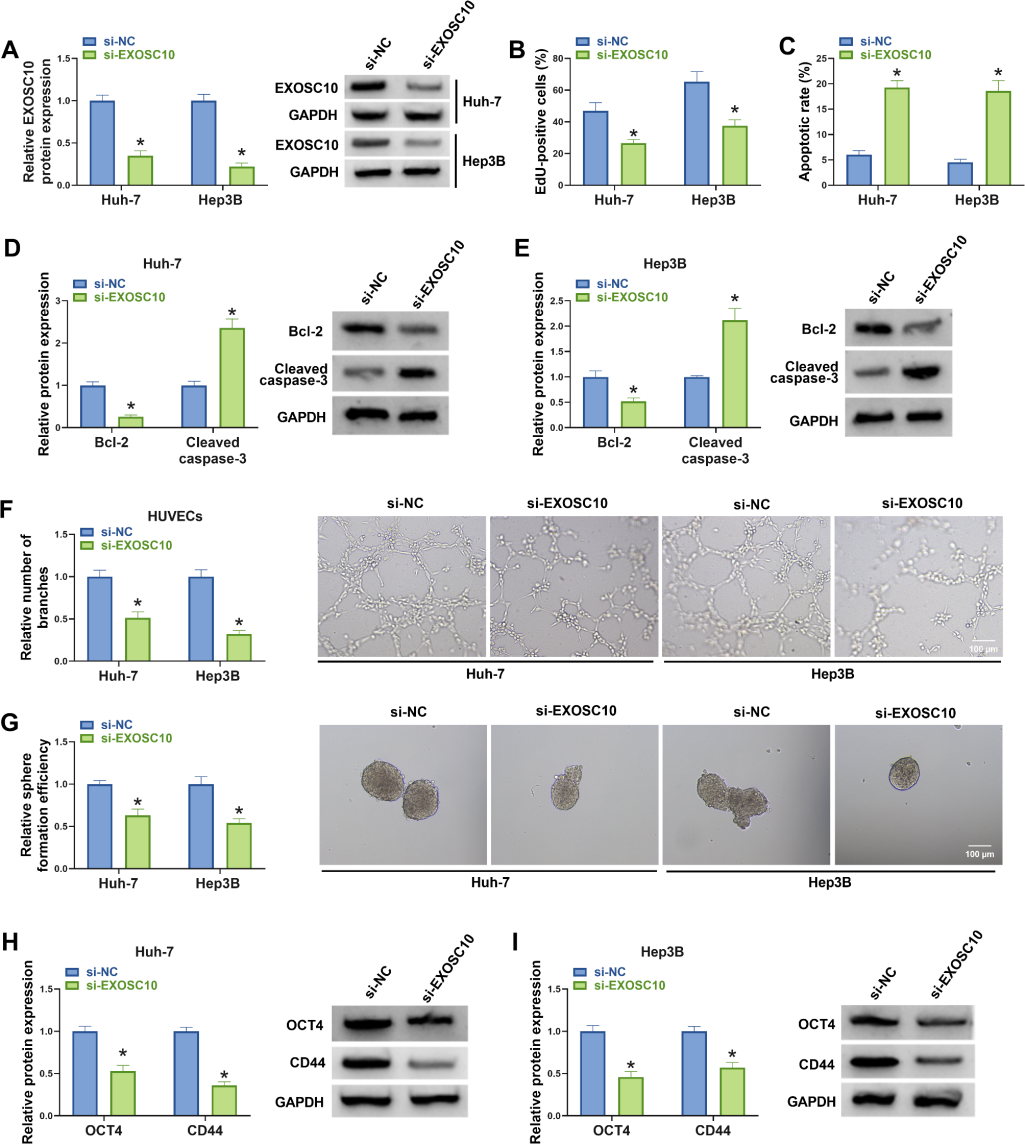
EXOSC10 silencing suppresses HCC cell proliferation, angiogenesis, and stemness. (**A-G**) Huh-7 and Hep3B cells were transfected with si-NC or si-EXOSC10. (**A**) Western blotting analysis for EXOSC10 expression in cells. (**B**) EdU assay for cell proliferation. (**C**) Flow cytometry for cell apoptosis. (**D, E**) Detection of Bcl-2 and Cleaved-caspase-3 protein levels by western blotting. (**F**) Analysis of capillary-like structures formed by HUVECs by tube formation assay. (**G**) Tumor sphere formation assay for sphere formation rate. (**H, I**) Detection of OCT4 and CD44 protein levels by western blotting. *P < 0.05

Subsequently, we probed the functions of EXOSC10 on HCC cells. Western blotting analysis showed si-EXOSC10 introduction markedly reduced EXOSC10 expression in HCC cells (Fig. 4A). In further function analyses, EXOSC10 silencing suppressed the proliferation (Fig. 4B) but induced apoptosis (Fig. 4C-E) in Huh-7 and Hep3B cells. Besides that, EXOSC10 deletion repressed the number of branches formed by HUVECs (Fig. 4F). The tumor sphere formation assay showed that EXOSC10 deletion declined the sphere formation rate in Huh-7 and Hep3B cells (Fig. 4G), as well as impaired the expression of OCT4 and CD44 proteins (Fig. 4H, I), indicating the inhibition of cancer cell stemness after EXOSC10 knockdown.

### E2F1 regulates HCC cell proliferation, angiogenesis, and stemness via EXOSC10

**Fig. 5:**
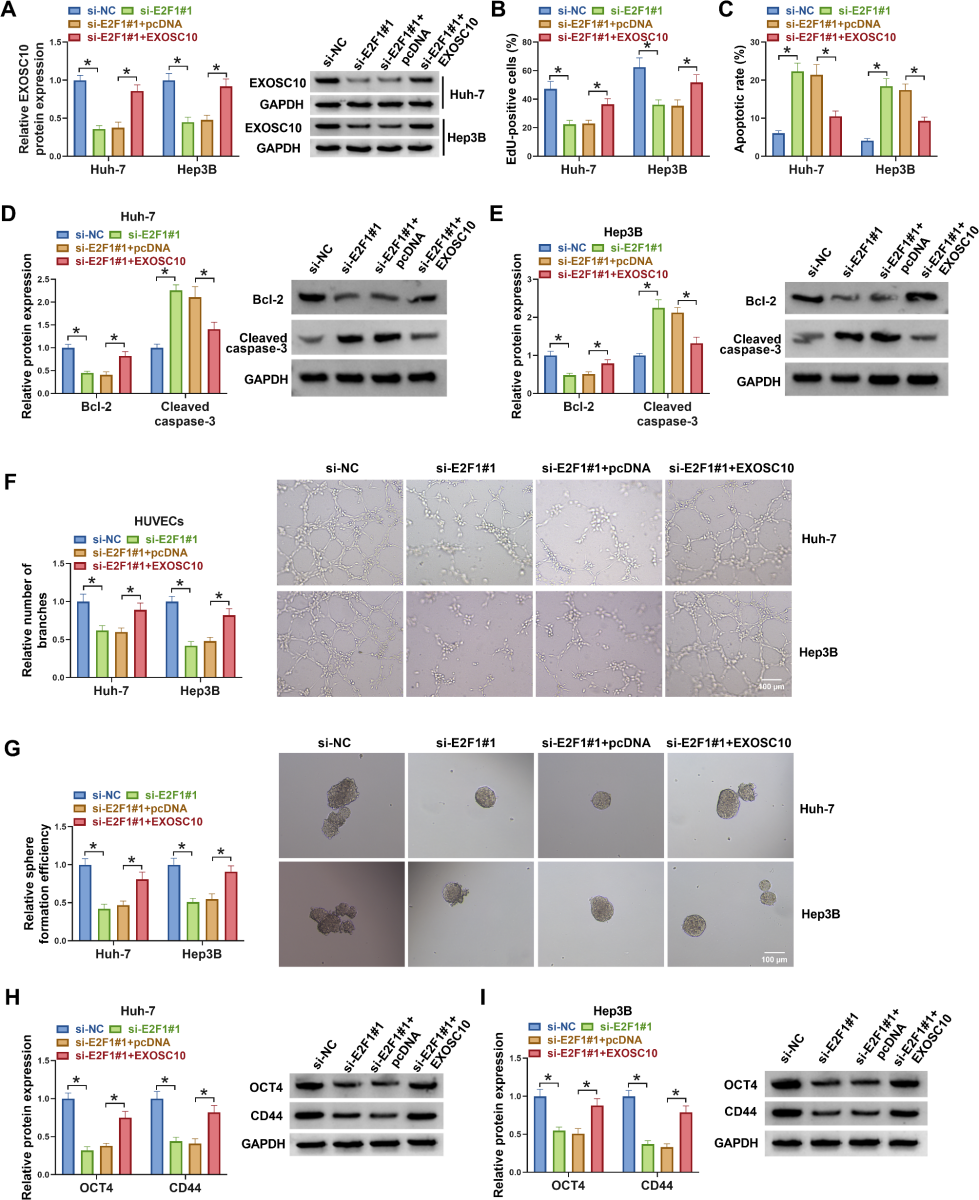
E2F1 regulates HCC cell proliferation, angiogenesis, and stemness via EXOSC10. (**A-G**) Huh-7 and Hep3B cells were transfected with si-E2F1#1 alone or si-E2F1#1 and EXOSC10. (**A**) Western blotting analysis for EXOSC10 expression in cells. (**B**) EdU assay for cell proliferation. (**C**) Flow cytometry for cell apoptosis. (**D, E**) Detection of Bcl-2 and Cleaved-caspase-3 protein levels by western blotting. (**F**) Analysis of capillary-like structures formed by HUVECs by tube formation assay. (**G**) Tumor sphere formation assay for sphere formation rate. (**H, I**) OCT4 and CD44 protein levels were assayed by western blotting. *P < 0.05

Next, we explored whether EXOSC10 was involved in the action of E2F1 on HCC cells. Huh-7 and Hep3B cells were transfected with si-E2F1#1 alone or si-E2F1#1 and EXOSC10. Expectedly, si-E2F1#1 introduction reduced EXOSC10 expression, which was rescued by EXOSC10 vectors in HCC cells (Fig. 5A). Functionally, we found EXOSC10 overexpression reversed E2F1 deficiency-induced proliferation inhibition (Fig. 5B), apoptosis enhancement (Fig. 5C), angiogenesis suppression (Fig. 5D-E), and cancer stemness impairment (Fig. 5G-I) in Huh-7 and Hep3B cells.

### E2F1 knockdown impedes HCC tumor growth by regulating EXOSC10 in vivo

**Fig. 6:**
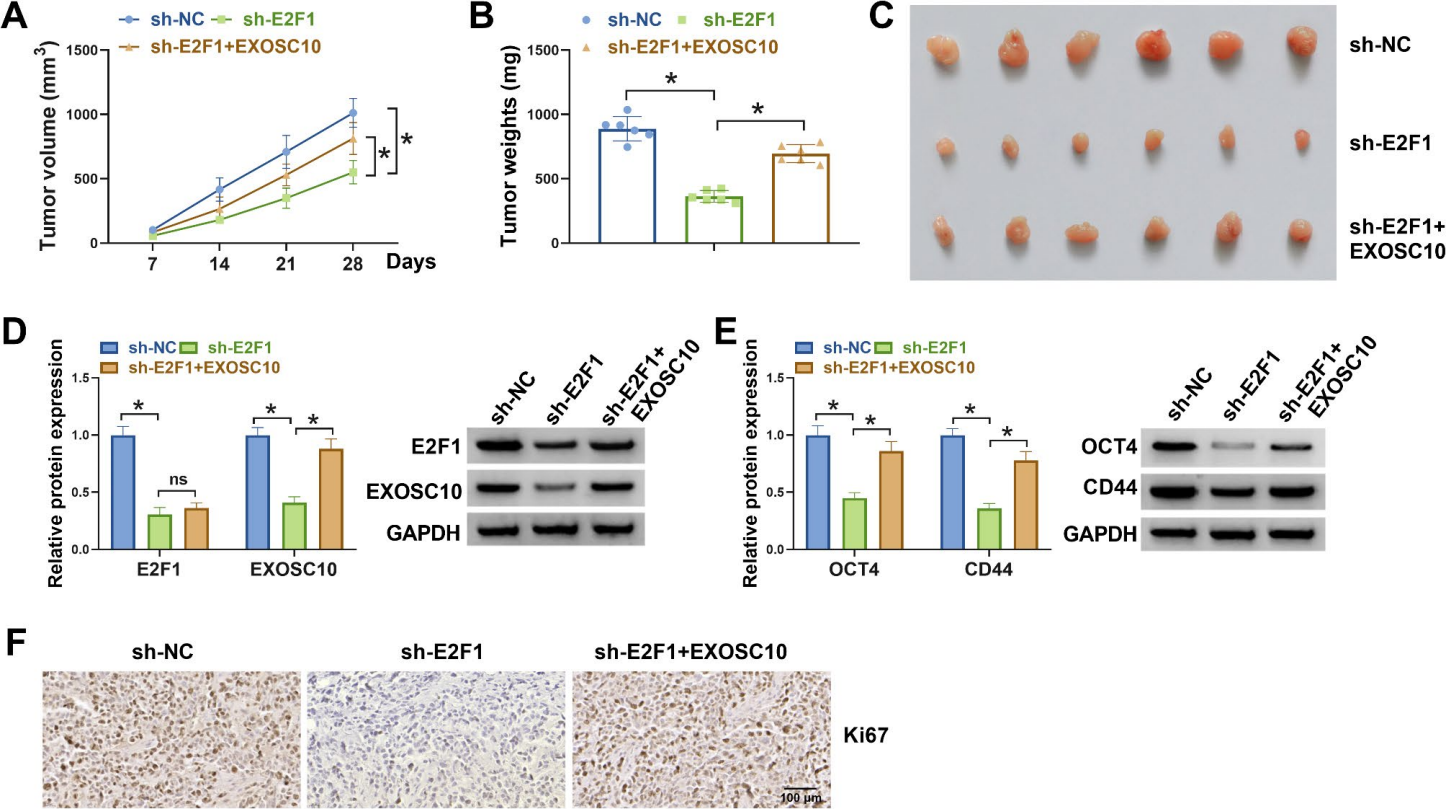
E2F1 knockdown impedes HCC tumor growth by regulating EXOSC10 in vivo. (**A-C**) The growth curve of tumors (**A**), the weight of xenograft tumors (**B**), and representative tumors (**C**). (**D, E**) Measurement of E2F1, EXOSC10, OCT4, and CD44 protein levels in xenograft tumors. (**F**) IHC analysis for Ki67 protein in xenograft tumors. *P < 0.05

Finally, we investigated the effects of E2F1 and EXOSC10 on HCC growth in vivo. A xenograft model was established. As exhibited in Fig. 6A-C, E2F1 knockdown was proved to suppress tumor growth, evidenced by smaller and lighter tumors in sh-E2F1 group, however, this suppression was abolished by EXOSC10 overexpression. Furthermore, E2F1 knockdown led to a decrease of E2F1 and EXOSC10 protein levels in xenografts, while the decreases of EXOSC10 protein mediated by E2F1 knockdown were reduced by EXOSC10 overexpression (Fig. 6D). In addition, OCT4 and CD44 protein levels were also decreased in xenografts of the sh-E2F1 group, but their expression levels were higher in xenografts of the sh-E2F1 + EXOSC10 group (Fig. 6E). IHC analysis further showed E2F1 knockdown suppressed KI67 protein expression in xenografts, which was rescued by EXOSC10 up-regulation (Fig. 6F).

## Discussion

This study showed a high expression of E2F1 in HCC, and high E2F1 expression was tightly related to poor outcomes in HCC patients. Functionally, E2F1 deficiency was proved to suppress HCC cell proliferation and angiopoiesis, as well as impaired cancer cell stemness in vitro and in vivo. Sustained and abnormal proliferation is a hallmark of cancer cells, operating not only in early tumorigenesis but also in cancer metastasis [23]. Angiogenesis, a process in which new blood vessels form in pre-existing vessels, is a critical event for cancer growth, diffusion and hematogenous metastasis, thus contributing to the development of diverse malignancies [24, 25]. Cancer cells need to acquire some stem cell-like properties, including capacities for self-renew and differentiation, to survive and adapt to ever-changing environments. That is stemness, which represents a critical mechanism for sustaining cancer progression [26, 27]. Thus, E2F1 siRNAs or shRNAs may be promising molecules for HCC therapy.

Thereafter, the downstream genes of E2F1 were investigated. We discovered that E2F1 induced EXOSC10 transcription in HCC cells. EXOSC10, predominantly located in cell nucleus, is a catalytic subunit of the multimeric exosome that possesses 3’-5’ exoribonuclease activity [28, 29]. EXOSC10 is the target of auto-antibodies produced in patients suffering from polymyositis/scleroderma overlap syndrome and has broad clinical importance [29, 30]. EXOSC10/Rrp6 is implicated in RNA processing and degradation, as well as DNA double-strand break repair and control of telomere maintenance [31, 32]. EXOSC10 is closely associated with the poor outcome [33] and immune infiltration in HCC, deletion of EXOSC10 eliminated the migration and growth of HCC cells via the p53 pathway [20]. Transforming growth factor-β (TGF-β) signaling functions diversely among different cell types in a context-dependent manner, and has been shown to play a key role in regulating cell survival, growth, proliferation, metabolism, differentiation, adhesion, migration, and immunity [34]. Moreover, Wang et al. suggested that EXOSC10 down-regulation effectively inhibited the TGF-β signaling pathway [35]. Herein, we found an increased expression of EXOSC10 in HCC, knockdown of EXOSC10 could impair HCC cell proliferation, angiopoiesis, and stemness. Moreover, EXOSC10 up-regulation reversed the anticancer effects of E2F1 deletion on HCC. Nevertheless, our research has some limitations. The downstream signaling pathways involved in EXOSC10 silencing should be further investigated. Secondly, whether there are other transcription factors that also regulate EXOSC10 or whether other transcription factors synergistically act with E2F1 on EXOSC10 still needs to be further explored. In addition, the subcutaneous xenograft used in our work may not fully mimic the natural HCC microenvironment, therefore, the reproducibility of some of our findings in natural disease state remains to be validated.

In conclusion, this study first confirmed that E2F1 up-regulated EXOSC10 expression by promoting its transcription, thereby enhancing HCC growth and cancer stemness. Currently, the RNA interference (RNAi) has been found to possess enormous potential for clinical purposes. Over the past 20 years, siRNA-based therapies have been actively developed, and siRNA agents, such as patisiran, lumasiran, and givosiran, have been approved by the FDA for disease therapy [36]. Thus, this research suggests a new insight into the development of E2F1 or EXOSC10 siRNA-based therapies for HCC.

## Electronic supplementary material

Below is the link to the electronic supplementary material.

Supplementary Material 1

Supplementary Material 2

## Data Availability

No datasets were generated or analysed during the current study.
