# Supplementary material for: E2F1-driven EXOSC10 transcription promotes hepatocellular carcinoma growth and stemness: a potential therapeutic target
Source: Hereditas. 2025 Apr 12;162:60. doi: 10.1186/s41065-025-00430-7 (PMC11992873; doi:10.1186/s41065-025-00430-7)
Supplement: Supplementary file 1 — Supplementary Material 1 [file 41065_2025_430_MOESM1_ESM.docx]

Table S1 The clinicopathological characteristics of HCC patients (N=66)

| Events | Number |
| --- | --- |
| Gender |  |
| Male | 38 (57.6%) |
| Female | 28 (42.4%) |
| Age |  |
| ≤ 50 | 25 (37.9%) |
| > 50 | 41 (62.1%) |
| HBsAg |  |
| Negative | 26 (39.4%) |
| Positive | 40 (60.6%) |
| Serum AFP(μg/L) |  |
| ≤ 400 | 37 (56.1%) |
| > 400 | 29 (43.9%) |
| Liver cirrhosis |  |
| Absence | 36 (54.5%) |
| Presence | 30 (45.5%) |
| Tumor size (cm) |  |
| ≤ 5 | 42 (63.6%) |
| > 5 | 24 (36.4%) |
| T stage |  |
| I+II | 43 (65.2%) |
| III+IV | 23 (34.8%) |
