# Supplementary figures and images for: E2F1-driven EXOSC10 transcription promotes hepatocellular carcinoma growth and stemness: a potential therapeutic target
Source: Hereditas. 2025 Apr 12;162:60. doi: 10.1186/s41065-025-00430-7 (PMC11992873; doi:10.1186/s41065-025-00430-7)

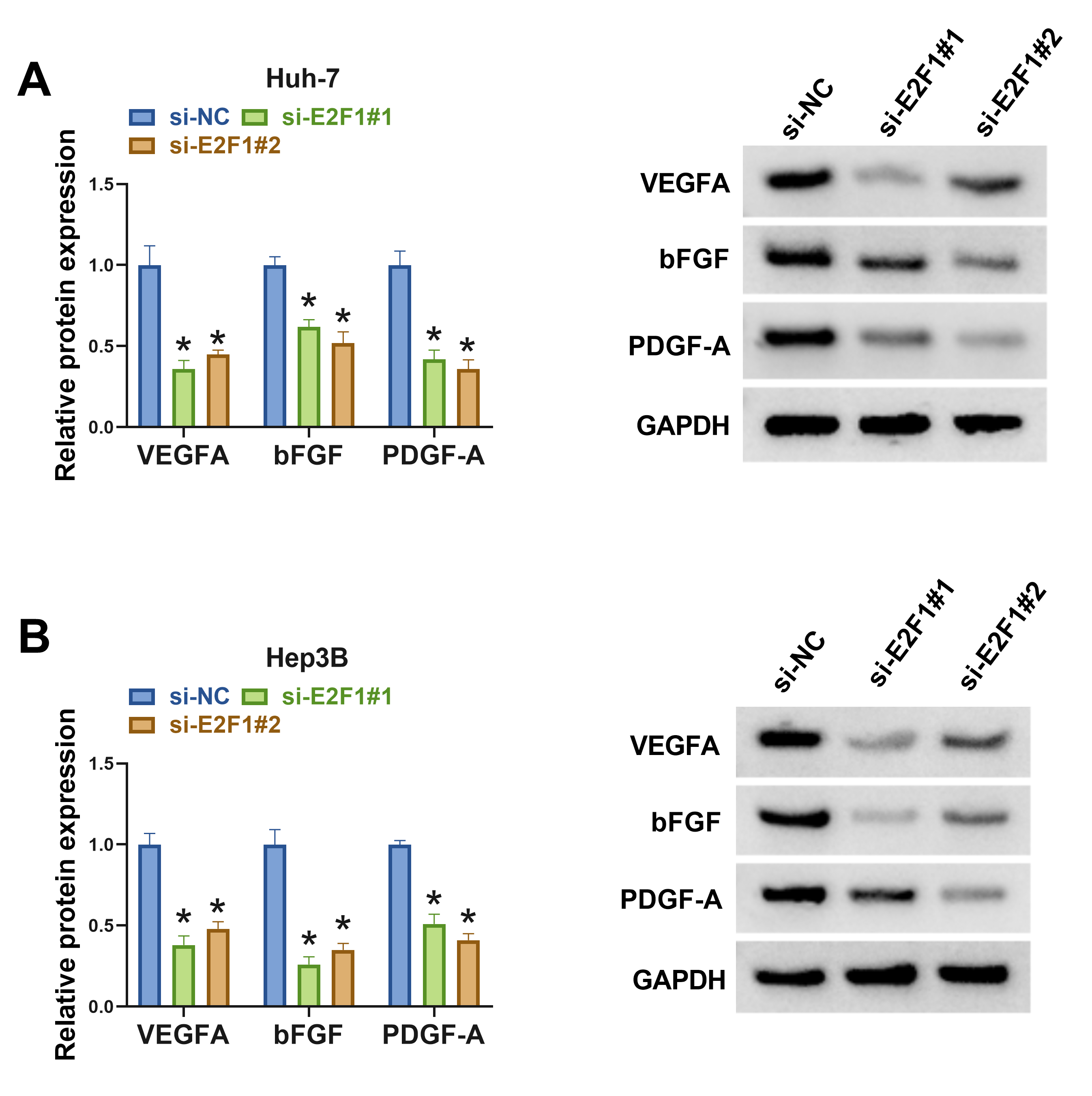

Supplement: Supplementary file 2 — Supplementary Material 2 [file 41065_2025_430_MOESM2_ESM.tif]
